# Effect of *Alchornea cordifolia* on Glycemic Indices of Varieties of Fufu Among Healthy Subjects

**DOI:** 10.1016/j.cdnut.2024.102076

**Published:** 2024-01-10

**Authors:** Eunice T Otoo, Marina A Tandoh, Felix C Mills-Robertson

**Affiliations:** 1 Department of Biochemistry and Biotechnology (Human Nutrition and Dietetics), College of Science, PMB, KNUST, Kumasi, Ghana; 2 Department of Biochemistry and Biotechnology (Microbiology), College of Science, PMB, KNUST, Kumasi, Ghana

**Keywords:** glycemic index, *Alchornea cordifolia*, phytochemical, blood glucose, carbohydrate, fufu

## Abstract

**Background:**

Glycemic index (GI) is a measure of the ability of carbohydrate food to raise blood glucose concentration. The GI of a food and its negative effects has caused an adverse increase in the prevalence of diabetes and other metabolic diseases.

**Objective:**

This study aimed to determine the effect of *Alchornea cordifolia* on glycemic indices of varieties of fufu.

**Methods:**

The research was a crossover experimental study involving 10 healthy individuals. A 50-g measure of pure glucose was served on 2 separate occasions and, subsequently, a measured amount of the test foods containing 50 g of available carbohydrates. The GI values were determined by the measure of the blood glucose concentrations of the subjects at fasting and after ingestion of the glucose and the test foods (fufu) within 2 h. Collection of capillary blood for blood glucose measurement started 30 min after consumption and was subsequently taken at 60, 90, and 120 min for both noncomposited and composited fufu GI determination. The phytoconstituents of the *A*. *cordifolia* were also determined.

**Results:**

For the noncomposited fufu, plantain fufu had the least glycemic response (46%), followed by cassava fufu (50%) and cassava–plantain fufu (53%); however, all were in the low-GI category. For the composited fufu, plantain fufu had the least response (12%), followed by cassava–plantain fufu (14%) and cassava fufu (14%), with all in the low-GI category. A multiple comparison of GI on the various foods by analysis of variance revealed a significant difference between the GI of cassava–plantain fufu and composite cassava–plantain fufu (*P* = 0.001); cassava fufu and composite cassava fufu (*P* = 0.004); and plantain fufu and composite plantain fufu (*P* = 0.006). The phytochemical screening of the *A*. *cordifolia* revealed the presence of flavonoids and tannins.

**Conclusions:**

Composited *A*. *cordifolia* fufu affects the glycemic response.

## Introduction

Carbohydrate is the main energy source in most diets consumed by humans, which make up approximately 40%–80% of the total calorie intake and act as a major aspect of the physiologic domain in the body concerning blood glucose concentration [1]. The glycemic index (GI) of a certain food is determined by factors, such as the carbohydrate nature, fiber content, carbohydrate food structure, and processes during preparation [[2], [3], [4]]. Some carbohydrate foods cause a quicker response from insulin than other carbohydrate foods [5], which is due to their differences in the degree of releasing glucose into the bloodstream concerning GIs [6]. Meals of the majority of Ghanaians are based on carbohydrates, and a lot of families plan their diets from it [7]. When one consumes carbohydrate-containing foods that are made up of sugars and starches, the body converts them (sugars and starches) into glucose that again enters the blood and causes a rise in the concentration of blood glucose [6]. According to epidemiologic evidence, blood glucose concentration rises at a faster rate after the consumption of certain types of carbohydrate meals [8]. The measurement of the blood glucose concentrations as influenced by a meal is known as the GI of that food [7].

The concept of GI, which is the associated capability of a carbohydrate food to raise the blood glucose concentration after consuming a meal, was generally proposed by Jenkins et al. [9]. Thus, high-GI foods are those which comprise carbohydrates that catabolize at a faster rate during the digestion process and become quickly absorbed into the bloodstream [10,11]. The GI can also be described as the area under the glucose response curve after consuming a carbohydrate (50 g) from a test food, which is divided by the area under the curve after consuming a carbohydrate (50 g) from a reference food [3]. Thus, the GI is a tool that helps compare foods that contain carbohydrates and compares how quickly these foods raise blood glucose concentrations. Hence, the higher the GI, the faster the food will raise the blood glucose concentration [12]. GI value shows how quickly glucose enters the blood after food ingestion, and causes an effect on the blood glucose concentration [13]. The longer the blood glucose remains raised, the extreme the chronic damage [14]. Foods with a high-GI increase an individual’s risk for certain health conditions linked with hyperinsulinemia, which includes diabetes mellitus [15], body weight problems, and cardiovascular disease [14].

*Alchornea cordifolia*, also called Christmas bush, is a known and largely used perennial evergreen dioecious shrub or small tree and a plant from the family Euphorbiaceae in the kingdom Plantae [16]. It is often found in Central Africa and grows up to 4–8 m tall and consists of many branches of simple leaves that are broad and oval as long as 10–28 cm and 6.5–16.5 cm wide [17]. It is usually found in forest zones and grows very well in damp environments throughout the year without irrigation, thus it is available every season for a year. Moroever, it contributes to the replenishing of the soil through its foliage droppings [18]. Although the content of *A*. *cordifolia* is high in indigestible fiber, it is also a good source of protein, potassium, calcium, and phosphorus [18]. *A*. *cordifolia* is generally regarded as a medicinal plant with its leaves mostly used but the stem, roots, and fruits are also considered medicinal, especially in the traditional setting [19,20]. The phytochemical constituents of *A*. *cordifolia* show the presence of secondary metabolites and their various therapeutic effect [21]. The secondary metabolites present include terpenoids, flavonoids, tannins, saponins, and steroid glycosides [17,21]. *A*. *cordifolia* is known for its antidiabetic, analgesic, anxiolytic, anti-inflammatory, spasmolytic, antibacterial, antimicrobial, antidiarrheal, antimalarial, and antioxidant properties [17]. Therefore, it is considered in the treatment of diseases and conditions, such as diabetes, high-blood pressure, hemorrhoids, cough, bronchitis, genital–urinary problems, female sterility, anemia, gastric ulcers, diarrhea, epilepsy, and tachycardia [17,[22], [23], [24], [25]]. The genus *Alchornea* has proven to possess good antioxidant properties [26].

The antioxidant activity of *A*. *cordifolia* is also correlated to the content of polyphenols (tannins and flavonoids) [27,28]. The antioxidants can act as reducing agents, thereby being able to oxidize themselves in a reaction and reducing the other chemicals involved with it [29]. The reducing effect of polyphenols acts by inhibiting glucose absorption in the gut by peripheral tissues, leading to a decrease in GI [30]. Some known polyphenols of *A*. *cordifolia* exhibit their reducing effect with the ability to lower glucose concentrations by delaying intestinal glucose absorption [31]. Thomford et al. [32] indicated in a study that the extraction from *A*. *cordifolia* decreased in blood glucose concentrations. Thus, despite the risk involved with high-GI food consumption, medicinal plants, such as *A*. *Cordifolia*, can be used as a preventive measure against what may arise [21].

## Methods

### Study area

Data were collected from 16 March 2021, to 8 April 2021, at the Clinical Analysis Laboratory (CAn Lab) of the Department of Biochemistry and Biotechnology, KNUST, Kumasi. The independent variable, the fufu, was prepared by the researcher and made from a sole carbohydrate food without any accompaniment to assess its effect on the glucose response of the individual. To ensure the desired result was achieved, the duration for consumption and the movements of the participants after consumption of the test foods were controlled.

The evaluation of the *A*. *cordifolia* leaves for its phytoconstituents was performed at the Clinical Microbiology Laboratory of the Department of Biochemistry and Biotechnology, KNUST.

### Study design

This research was a crossover experimental study. The participants involved in the study received all test treatments that were being investigated at different times [33,34]. Initially, participants initially were asked to consume 50 g of glucose mixed in 200 mL of water as reference food, and capillary blood was drawn for blood glucose measurements at 30, 60, 90, and 120 min following consumption for 2 separate testing days. Subsequently, the participants after taking the reference food were given the test food comprising varieties of fufu either composited or noncomposited on different testing days. After consumption of each of the test foods, capillary blood was redrawn for blood glucose measure at 30, 60, 90, and 120 min. The reference food and each of the test foods involved were administered separately on different testing days. Participants were made to undergo 10–14 h of fasting between the last meal of the previous day to the morning of the testing day. This contributed to a small study population and again eliminated variability among individuals.

### Study population

Ten healthy persons were purposively recruited for the clinical test, comprising 7 males and 3 females in sequence to their approval. Ten subjects were selected for the study based on the recommendation by FAO/WHO [35] for GI determination, which says the test can be repeated in >6 subjects, and on other studies by Wolever et al. [36] and Brouns et al. [37], who also recommended that precisely 10 subjects will yield accurate results.

### Ethics

Approval for the research was given by the Committee of Human Research Publication and Ethics of Komfo Anokye Teaching Hospital/School of Medical Sciences, KNUST, Kumasi (CHRPE/AP/119/21).

### Preparation of test meals

The foods were cooked on separate days but went through the same procedure. The foods (cassava and plantain) were peeled and washed thoroughly. Thereafter, they were cut into smaller portions and boiled, but for the composite test meals, they were boiled with the leaves of *A*. *cordifolia*. After the foods were properly cooked, they were pounded until a desired fufu paste was achieved and divided into measured portion sizes that were served in cleaned containers. For the composite food, 4 kg weight of the peeled foods (cassava and plantain) were cooked with 25.2 g shade-dried leaves of the *A*. *cordifolia* in ∼3 L of water.

For the soup, the garden eggs, tomatoes, onions, and pepper were washed, boiled, and blended after they were well cooked. The blended vegetables were poured back into the saucepan and the desired amount of water was added until the quantity was achieved. After they were half-cooked, the smoked salmon and a pinch of salt (for taste) were also added and made to boil until fully cooked.

The containers containing the specific quantity of fufu with 50 g available carbohydrate portions were sent to the study site and served with 3 soup ladles of light soup, and 3 matchbox of salmon for the participants to consume.

### Preparation of crude extract from leaves

*A. cordifolia* leaves were harvested from the Physic garden of the Department of Herbal Medicine, KNUST. The fresh leaves were washed several times under running tap water and once with sterile water and shade dried at room temperature; afterward, the leaves were stored in airtight bags [38].

### Ethanol extract preparation from leaves

For the ethanol extract preparation, 100 g of the pulverized plant materials were cold-macerated for 3 d with 1 L of 70% ethanol for 3 d. The ethanol extracts were filtered through 2-fold muslin cloth proceeded by Whatman No.1 paper. Then, the filtrate was concentrated at 65 °C in a rotatory evaporator. The concentrate retrieve was altered with water and freeze dried for 24 h to eliminate the water. Finally, it was stored in an airtight container and refrigerated until needed [38].

### Aqueous extract preparation from leaves

The aqueous extract was prepared according to the methods suggested by Mills-Robertson et al. [39]. In brief, the aqueous extract of the leaves was achieved by soaking the leaves in distilled water for 30 min before boiling them in distilled water for 30 min. Then, the hot mixture was allowed to simmer for an extra 30 min under reduced temperature, cooled, and freeze dried to obtain a powdered product. The resulting powder was stored in an airtight container and kept in a desiccator until needed [39].

### Phytochemical screening

Phytochemicals was determined using the standard methods of Edeoga et al. [40] on both aqueous and ethanol extract of the leaves. The test included Wagner test for alkaloids, foam test for saponins, Braymer test for tannins, alkaline reagent test for flavonoids, Salkowki test for terpenoids, ferric chloride test for phenols, and Keller Kelliani test for cardiac glycosides.

#### Test for alkaloids (Wagner reagent test)

A 2 mL of the leaf extract was treated with 3 drops of 1% hydrochloric acid, heated at 60 °C and cooled. A few drops of Wagner reagent were added, and the presence of a reddish brown precipitate showed the presence of alkaloids.

#### Test for saponins (foam test)

A volume of 6 mL of distilled water was added to 2 mL of the leaf extract and shaken vigorously. The formation of constant foam indicated the presence of saponins.

#### Test for terpenoids (Salkowki test)

A volume of 1 mL of chloroform was added to 2 mL of the extract and in sequence by a few drops of concentrated sulfuric acid, and the prompt presence of a reddish brown precipitate indicated the presence of terpenoids.

#### Test for tannins (Braymer test)

A few drops of 10% FeCl_3_ solution were added to 2 mL of the leaf extract, and the formation of a deep green coloration marked the presence of tannins in the leaf extracts.

#### Test for flavonoids (alkaline reagent test)

A volume of 2 mL of the extract was treated with a few drops of 20% sodium hydroxide solution, and the formation of an intense yellow color that turned colorless after adding dilute hydrochloric acid indicated the presence of flavonoids.

#### Test for cardiac glycosides (Keller Kelliani test)

A volume of 5 mL of the leaf extract was treated with 2 mL of glacial acetic acid and a drop of FeCl_3_ solution and was attentively controlled with 1 mL of concentrated sulfuric acid. A brown ring at the interface marked the presence of cardiac glycosides.

#### Test for phenols (ferric chloride test)

A volume of 2 mL of the extract was treated with aqueous 5% FeCl_3_ and the formation of a deep blue or black color showed the presence of phenols.

### Data collection

The participants were required to provide some basic information, such as sex, residential address, waist circumference (WC), ethnicity, telephone number, occupation, and the time of last meal on a semistructured questionnaire given to participants.

### Study procedures

The participants were asked to fast for 10–14 h intervals between the last meal eaten of the previous day to the morning of the testing and to report at the Clinical Analysis Laboratory (CAnlab) premises by 7:30. Both the reference and test foods trials took place at the same venue and with the same reporting time.

A bathroom scale was used to measure the weight of the participants without shoes. A stadiometer was also used to measure their height in an upright position on their arrival and a measuring tape to measure WC. The height, weight, and WC measurements obtained were used for the analysis.

### Fasting blood glucose

Capillary blood was drawn from every participant to assay for the fasting blood glucose (FBG) using a ONE TOUCH select glucometer to affirm that the 10–14 h of fasting was observed by each of the participants. The process was performed as follows: the middle finger on the left hand of the participants was wiped clean with a piece of cotton and pricked with a lancet to draw a drop of capillary blood with a strip inserted in a glucometer, and glucose concentration recorded in millimoles per liter.

### Oral glucose tolerance test

Each of the participants was given a glucose solution, the reference food that was prepared using 50 g of glucose and 200 mL of water, and was asked to drink within 5 min using a stopwatch. The time at which each of the participants started to take in the glucose solution was recorded and 30 mins from the time participants started to take the glucose solution, a capillary blood was drawn from the participants and assayed for glucose concentration. In addition, blood samples were taken from the participants at 60, 90, and 120 min and assayed for the glucose concentration in millimoles per liter.

During the period of the testing, participants were asked not to leave the premises to ensure low-physical activity that may not interrupt the testing. Before the start of the activity, a dietary recall was conducted. Again, after the blood samples had been taken for 2 h, the participants were given a malt drink with a biscuit as an incentive and were informed of the subsequent testing days and reminded of the rules and regulations that govern their participation in the study.

### Test foods

Just as with the reference food, the participants were asked to fast for 10–14 h before the morning of testing day, with the same reporting time and venue. A dietary recall was conducted and recorded, and exactly 2 d after the reference food was given, the first test food was also given. To confirm the 10–14 h fasting, FBG was measured by a capillary blood drawn from every participant using ONE TOUCH select glucometer as described earlier.

For the procedure, an orange-sized cassava–plantain combination fufu with 3 soup ladles of light soup and 3 matchbox of salmon were given to each participant. The start time for every participant was recorded, and they were asked to finish the food within 10 min. The initial blood sample was taken 30 min from when the eating started, and subsequently, blood samples were taken at 60, 90, and 120 min, and the glucose concentrations were recorded.

Same procedure was followed on other testing days, at the same venue and reporting time. Capillary blood was assayed for FBG and recorded before any of the test foods were given. Test foods included cassava–plantain fufu, plantain fufu, and cassava fufu. After the general activity, participants were given malt and biscuits as an incentive before they left the premises.

### Data analysis

The glucose response curves were plotted with the GraphPad Prism software, version 5. The Incremental Area Under the glucose response Curves (IAUCs) was calculated by using the trapezoid rule recommended by the FAO/WHO [35]. All GIs that were 2 SD either more or less the mean GI value for a given test were ignored [36]. The Incremental Area Under the glucose response Curve (IAUC) for each test food was expressed in a percentage of the mean IAUC of the glucose of the reference food used. The GI of each test food was calculated as the mean GI obtained from each of the participants in the study.

The IAUC and GI were calculated using Excel, and data were presented as means and SDs. IBM Statistical Package for Social Sciences software, version 20, was used to analyze the data, and a *P* value of <5% at a 95% confidence level was considered statistically significant for the analyses. Values were analyzed using the 1-way analysis of variance.

## Results

The anthropometric parameters, such as weight, height, BMI, age, and WC were measured to determine the health status of the subjects (Table 1). The study subjects were 7 males and 3 females, with a mean age of 23.70 y, mean weight of 69.99 kg, mean height of 1.68 m, mean BMI of 25.07 kg/m^2^, and mean WC of 80.18 cm.

**TABLE 1:** Characteristics of the study subjects

	Age (y)	Weight (kg)	Height (m)	BMI (kg/m^2^)	Waist circumference (cm)
Mean	23.70 ± 0.98	69.99 ± 3.08	1.68 ± 0.03	25.07 ± 1.24	80.18 ± 2.67
Median	23	69.30	1.7	24.45	78.05
SD	3.09	9.75	0.08	3.33	8.45

The nutrient analysis of the various foods that were studied are tabulated in Table 2. The calculations were made based on 100-g portions. The available carbohydrates in the fufu was calculated by proximate analysis [41].

**TABLE 2:** Nutrient content of test meals

Test meal	Amount (g)	Protein (g)	Fat (g)	Total CHO (g)	Dietary fiber (g)	Energy (kcal)	Available CHO (g)
Fufu	144	1.76	0.36	52.39	2.88	214.56	50
Light soup	459	2.89	0.60	17.30	5.51	73.44	—
Salmon	279	51.00	12.1	0	0	326.43	—

The GI values from the measurement of the test foods are summarized in Table 3. The standard food (glucose) had a GI value of 100%. The cassava–plantain combination fufu had the highest GI value (53%) compared with individual staple foods, with the plantain fufu having the lowest GI value (46%). However, the GI values of all test foods were low according to the GI classification. The GI of the test foods was classified as either low, medium, or high as per the following: GI values ≤55, low; 56–69, medium; and ≥70, high. Table 3 presents the GI of the test foods and their classification, SE, and minimum and maximum values at 95% confidence.

**TABLE 3:** Glycemic index of the staples

Food item	At 95% CI			
	GI min (%)	GI max (%)	GI (%) SEM	GI Class
Glucose	100	100	100	High
Cassava–plantain fufu	23.58	79.16	53.33 ± 4.91	Low
Cassava fufu	20.37	82.91	50.97 ± 6.18	Low
Plantain fufu	0.00	98.94	46.90 ± 10.17	Low

Abbreviation: SEM, standard error of the mean.

The GI values from the measurement of the composited test foods are tabulated in Table 4. The GI of cassava–plantain composite fufu was 14%, that of composite cassava fufu was 14%, and that of plantain fufu was 12%. All composited test foods had low GI by the GI classification.

**TABLE 4:** Glycemic index of composited staples

Food item	At 95% CI			
	GI min (%)	GI max (%)	GI (%) SEM	GI Class
Glucose	100	100	100 ± 0.00	High
Cassava–plantain fufu	0.50	67.63	14.45 ± 6.57	Low
Cassava fufu	0.68	70.42	14.89 ± 6.47	Low
Plantain fufu	1.12	25.03	12.14 ± 2.71	Low

Abbreviation: Glycemic Index; SEM, standard error of the mean.

There was a significant difference between the IAUC of the reference food and the IAUC of the test food (*P* = 0.014) (Table 5). The IAUC is the area under the curve from the reference food and test foods. The trapezoid rule was used in the calculation.

**TABLE 5:** Incremental area under the curve of the test (normal and composite) and reference foods by the study subjects

	Glucose	Cassava–plantain fufu	Cassava fufu	Plantain fufu	Composite cassava–plantain fufu	Composite cassava fufu	Composite plantain fufu
Mean	101.13 ± 14.84	50.87 ± 7.08	49.2 ± 7.87	41.28 ± 7.30	14.28 ± 6.74	10.60 ± 3.51	11.19 ± 2.42
SD	46.94	22.39	24.88	23.08	21.32	11.10	7.65
Range	113.77	70.86	70.29	76.00	61.45	37.58	22.16
Minimum	51.23	23.85	16.71	0.00	0.35	1.13	0.72
Maximum	165.00	94.71	87.00	76.00	61.80	38.70	22.88

The aqueous and ethanol extracts of the *A*. *cordifolia* leaves were subjected to phytochemical screening (Table 6). The results showed the presence of alkaloids, flavonoids, phenols, saponins, tannins, and terpenoids but absence of cardiac glycosides in both aqueous and ethanol extracts.

**TABLE 6:** Phytoconstituents of *A*. *cordifolia*

Phytochemicals	Aqueous extract of *A*. *cordifolia*	Ethanol extract of *A*. *cordifolia*
Alkaloids	+	+
Cardiac glycosides	−	−
Flavonoids	+	+
Phenols	+	+
Saponins	+	+
Tannins	+	+
Terpenoids	+	+

**FIGURE 1:**
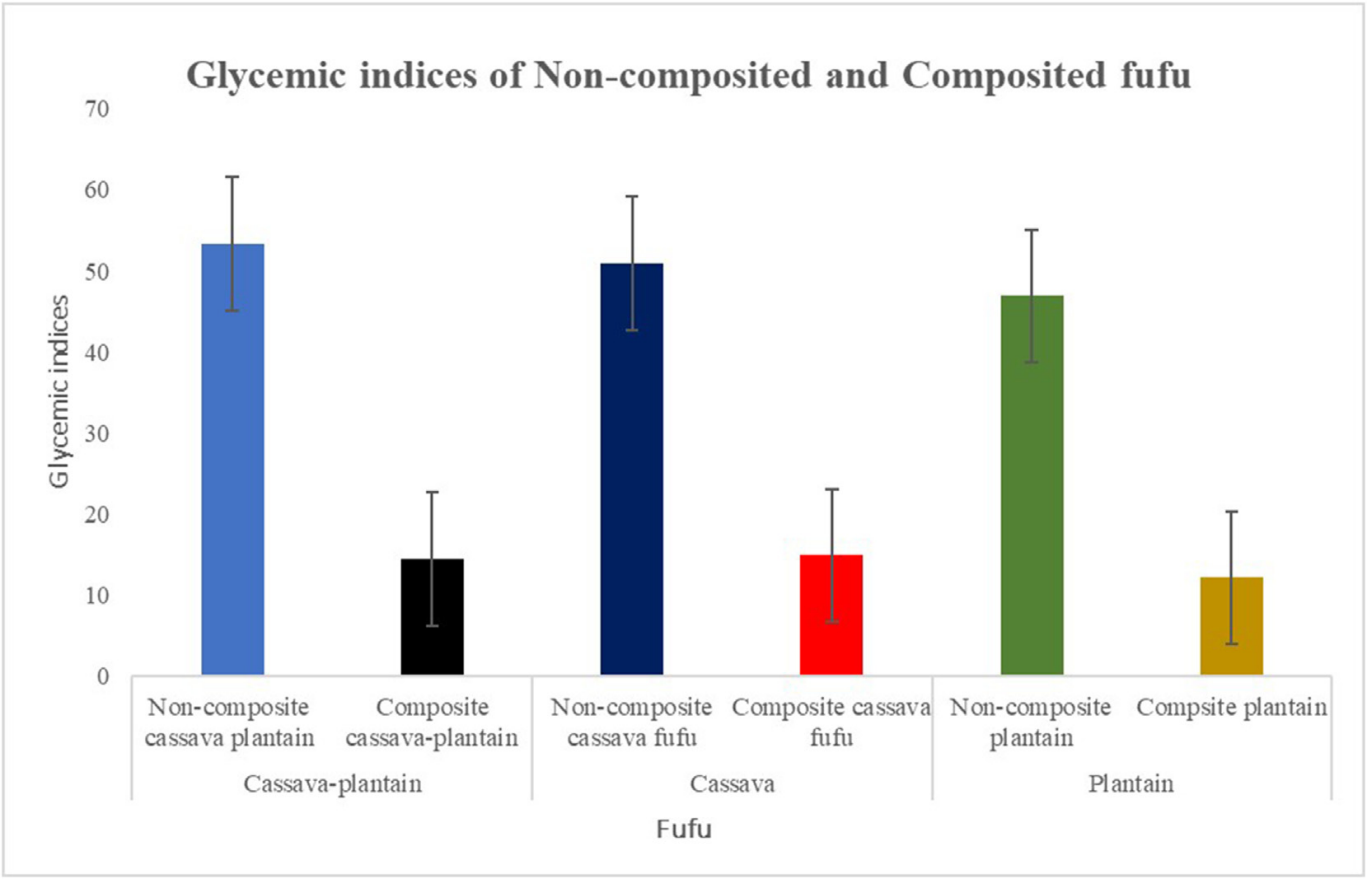
Glycemic indices of noncomposited and composited staple fufu.

A graphical representation comparing the noncomposite fufu to the composite fufu is presented in Figure 1. The GI of composite cassava–plantain fufu (14%) was significantly lower than that of the noncomposite cassava–plantain fufu (53%) (*P* = 0.001). Moreover, the GI of the composite cassava fufu (14%) was significantly lower than that of the noncomposite cassava fufu (14%) (*P* = 0.004), and that of the composite plantain fufu (12%) was also significantly lower than the GI of noncomposite plantain fufu (46%) (*P* = 0.006).

**FIGURE 2:**
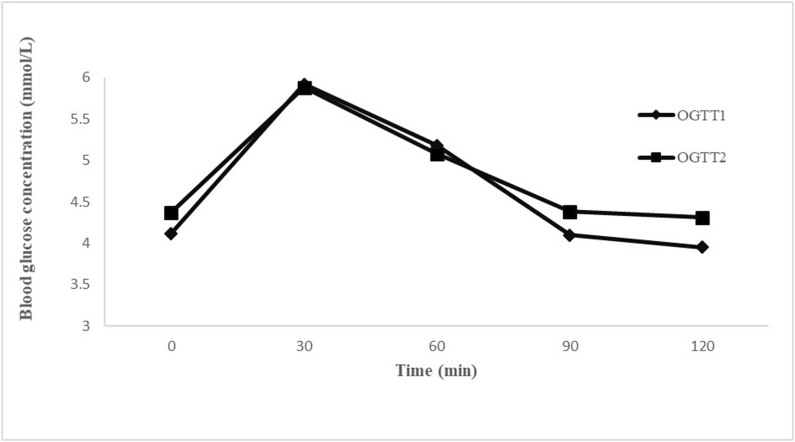
Mean glycaemic response obtained by 50-g glucose in duplicate. OGTT, oral glucose tolerance test.

As shown in Figure 2, the oral glucose tolerance test (OGTT) was done in duplicate. The mean peak of postprandial glucose in the subjects after they consumed the reference food was observed at the 30th min (OGTT 1 = 5.92; OGTT 2 = 5.88) from the ingestion of glucose, which declined at the 120th min (OGTT 1 = 3.95; OGTT 2 = 4.31).

**FIGURE 3:**
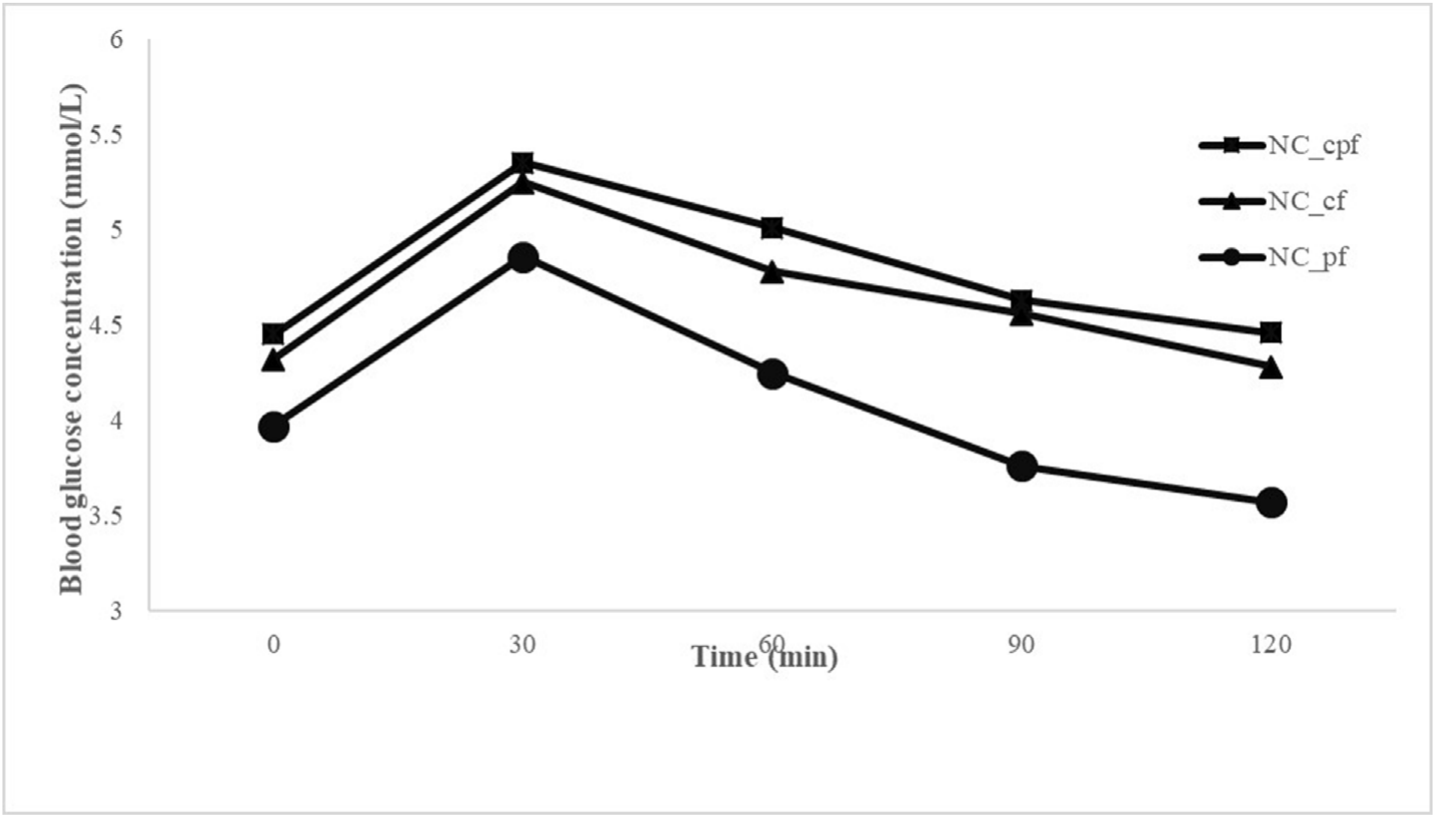
Mean glycemic responses obtained by the study subjects after consumption of 50 g available carbohydrate portions of different noncomposited samples. NC_cf, noncomposite cassava fufu; NC_cpf, noncomposite cassava–plantain fufu; NC_pf, noncomposite plantain fufu.

As shown in Figure 3 the mean peak of postprandial glucose obtained from the subjects after the consumption of the noncomposited fufu at 30 min was 5.35 for the noncomposited cassava–plantain fufu, 5.25 for the noncomposited cassava fufu, and 4.86 for the noncomposited plantain fufu from the ingestion of glucose. The decline at 120 min was 4.46 for the noncomposited cassava–plantain fufu, 4.28 for the noncomposited cassava fufu, and 3.57 for the noncomposited plantain fufu.

**FIGURE 4:**
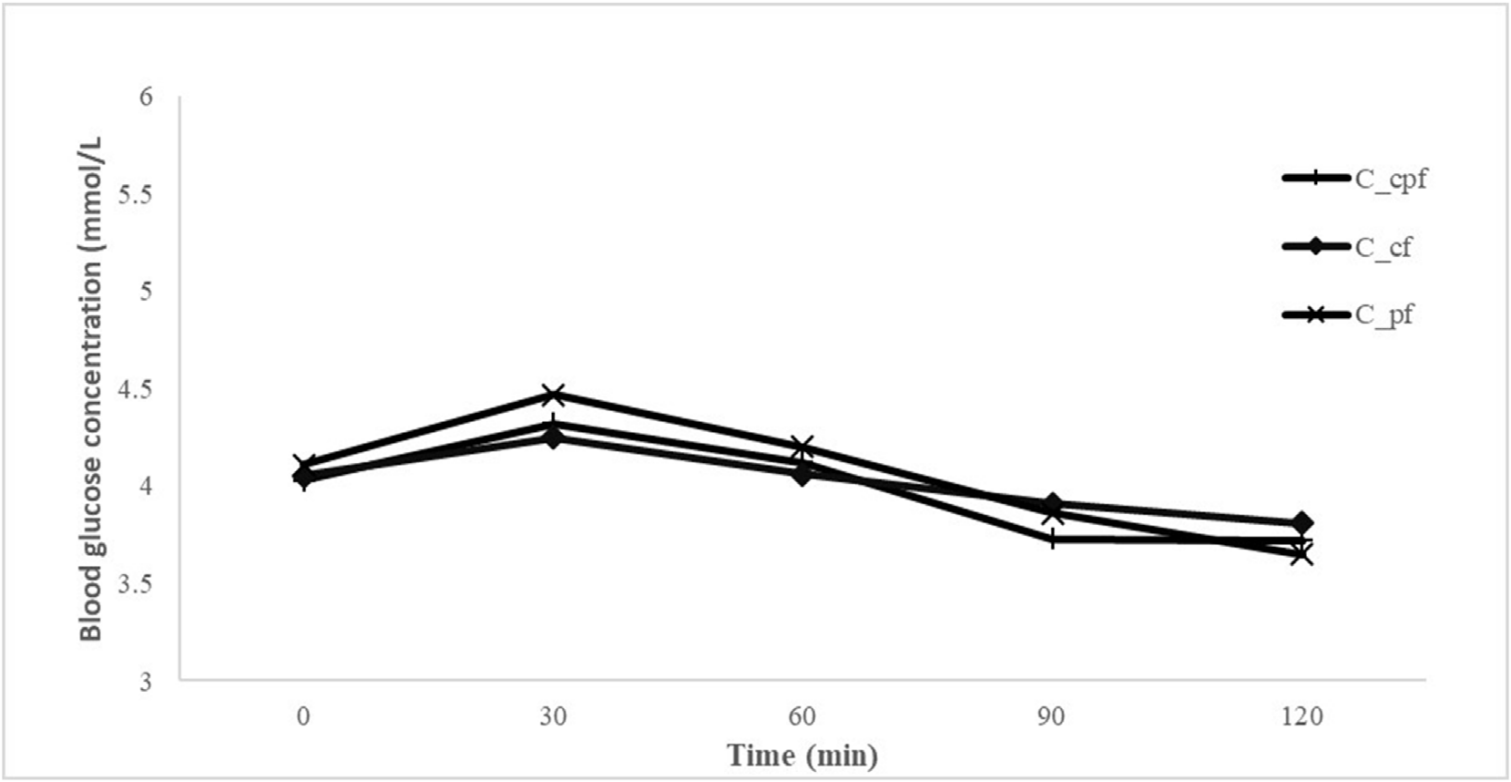
Mean glycemic responses obtained by the study subjects after consumption of 50 g available carbohydrate portions of different composited samples. C_cf, composite cassava fufu; C_cpf, composite cassava–plantain fufu; C_pf, composite plantain fufu.

Figure 4 shows the composited fufu test for the 3 varieties of fufu. The mean peak of postprandial glucose obtained from the subjects after the consumption of the composited fufu observed at 30 min was 5.35 for the composited cassava–plantain fufu, 5.25 for the composited cassava fufu, and 4.86 for the composited plantain fufu from the ingestion of glucose, and the decline at 120 min was 5.35, 5.25, and 4.86, respectively.

**FIGURE 5:**
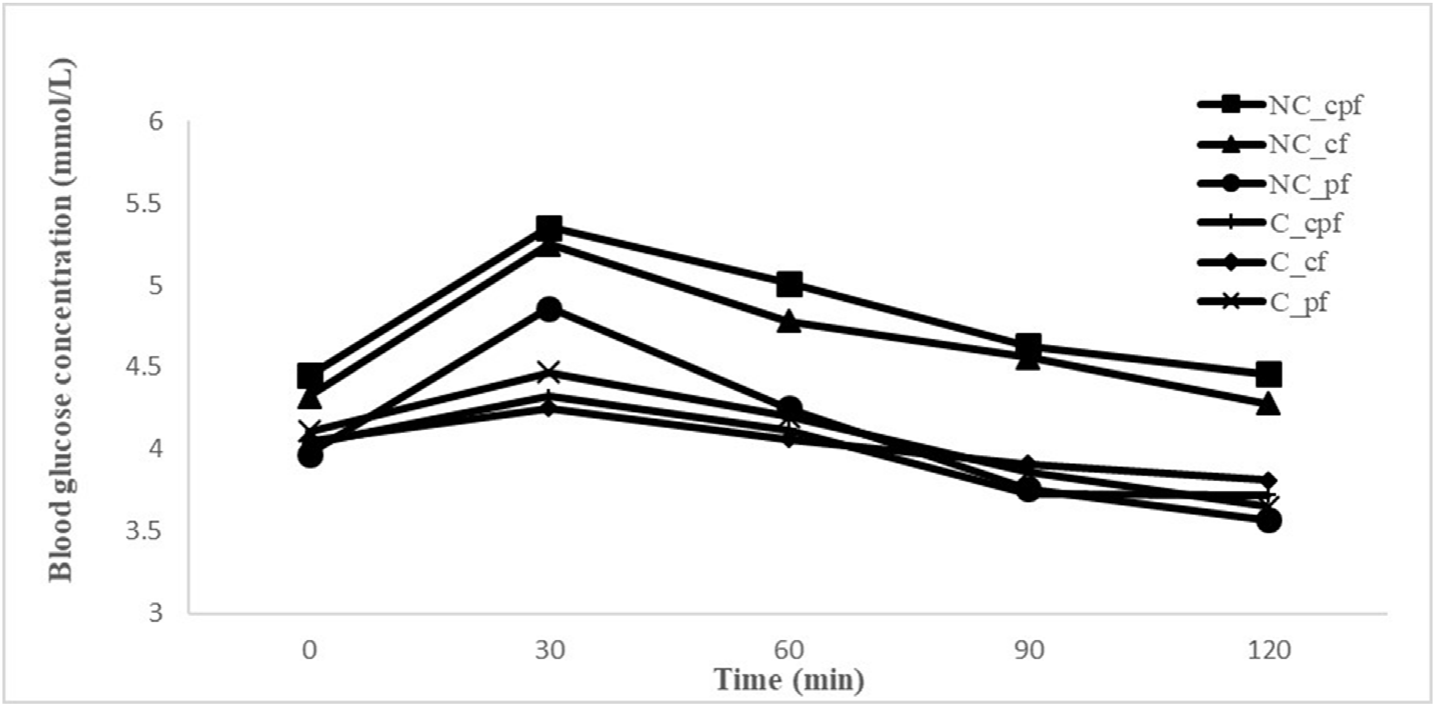
Mean blood glucose responses of the subjects obtained by the 50-g available carbohydrate portions of all varieties of fufu, both the normal and the composited. C_cf, composite cassava fufu; C_cpf, composite cassava–plantain fufu; C_pf, composite plantain fufu; NC_cf, noncomposite cassava fufu; NC_cpf, noncomposite cassava–plantain fufu; NC_pf, noncomposite plantain fufu.

Figure 5 shows the comparison of noncomposited and composited fufu GIs. The mean peak of postprandial glucose in the subjects after they consumed the meals was observed at 30 min and the decline at 120 min in all test foods. The composited fufu showed lower GI than the noncomposited fufu (Figure 5). The plantain fufu showed the lowest GI in both noncomposited (3.57) and composited (3.65) forms at 120 min, being more in the noncomposited form (3.57).

## Discussion

Most people with metabolic disorders, especially those with prediabetes and diabetes, restrict themselves from consuming certain foods, such as fufu, with the fear of the food influencing their blood glucose concentration detrimentally. Fortunately, the classification of carbohydrate foods, according to their glycemic responses, has provided knowledge on carbohydrate foods not suitable for persons with metabolic disorders and those deemed suitable. This study assessed the effect of *A*. *cordifolia* on the GIs of varieties of *A*. *cordifolia*–composited fufu, a delicacy in Ghana.

In this study, all 3 varieties of the pounded fufu, both noncomposited and composited, had low GIs. The GIs of all noncomposited fufu ranged from 46% to 53%. These figures for the noncomposited fufu, compared with the results from the studies by Adu-Gyamfi [42] and Eli-cophie et al. [7], were in the same range and, thus, recorded a low GI. In the case of the *A*. *cordifolia*–composited fufu, to our knowledge, this is the first study to be conducted in Ghana.

From the study, *A*. *cordifolia* on fufu had a high-magnitude effect on the glycemic response than the noncomposited fufu. Although both forms of fufu recorded low GIs, the *A*. *cordifolia*–composited fufu recorded the lowest GI values for all fufu varieties.

Regarding the action of *A*. *cordifolia* on the fufu, numerous studies on GI determination revealed some factors that affect the outcomes of the GI of foods, such as the fiber content of the food and the cooking method with associated temperature among others [7,11,43]. Fiber is known to influence glycemic responses; thus, the presence of fiber in both plantain and cassava might have contributed to the low-GI values of both the noncomposited and the composited fufu. The fiber content of freshly harvested cassava is 1.8 g and that of plantain is 3.06 g; however, all are reduced in their processed form [44]. Mann et al. [45] confirmed in a study that a diet rich in fiber slows down digestion, which in turn slows down the absorption of glucose into the bloodstream. Thus, in this study, the high amounts of fiber might have contributed to the postprandial glucose (low GI) response of participants, especially to plantain fufu because it has comparatively higher fiber content. Fufu is prepared by peeling, washing, heating, and cooling before pounding into soft balls [46]. The temperature of the various processes associated with the preparation of the fufu may somehow affect the food’s starch digestibility and, thus, affect glycemic response [47]. In other words, as the foods are exposed to various temperatures, the involved processes with higher temperatures cause the structure of the starch to change, hence leading to high digestibility. Foster-Powell et al. [8] revealed in a study that the processing methods of foods affect their GIs. For instance, the boiling of the staple food to make fufu can lead to some degree of starch gelatinization by softening the starch, thereby increasing the rate of digestion and the absorption of glucose. A study reported that combining foods to make a meal significantly affects the GI of the food [48]. In this study, the test foods were served with light soup and salmon fish, and the salmon, being a good source of fat and protein, may have led to the test foods having a low GI. Fat slows the rate at which dietary carbohydrates are digested in the intestines [43]. Moreover, Eleazu [49] in a study revealed that foods that contain protein increase insulin secretion and could contribute to the reason the starch in such foods is not easily hydrolyzed, contributing to the food’s low GI. The effect of digestion is also known to affect the GI of carbohydrate foods. For instance, in carbohydrate digestion, chewing is necessary to aid the rate of digestion and absorption [1,50]. The chewing breaks down large food particles into small particles in the mouth and it increases the surface area of the food for salivary amylase to act on it causing the breakdown of the complex carbohydrate before it enters the stomach for further digestion. In the case of fufu, it is not chewed in the mouth before entering the stomach because of the direct swallow to the stomach; hence, there is not enough surface area for the action of salivary amylase, and digestion may be slowed down in the small intestine, leading to low GI of the foods. Plantain has a high content of retrograded starch (R3-resistant starch) that is less susceptible to enzymatic breakdown [51], further lowering its glycemic response. In addition, the high-resistant starch content of plantain, the R2-resistant starch [52], might have contributed to the lowered glycemic response of the plantain fufu compared with that of the cassava–plantain fufu and the cassava fufu in this study. The presence of resistant starch and slowly digestible starch decreases the GI of plantain or cassava–plantain fufu. However, the low-GI values of the plantain or cassava–plantain fufu cannot be attributed entirely to the contributions from resistant or slowly digestible starches [53]. The GI of cassava varies greatly depending on the variety, growing conditions, and the nature and proportions of starch in the food. A study conducted revealed that the nature and proportion of starch in a food influence the rate of digestion and the glycemic response of the food [8].

In the case of *A*. *cordifolia* on the fufu, the GIs ranged from 12% to 14%, which was very low than those of the noncomposited fufu. The very low GIs of the composited fufu may be attributed to the effects of *A*. *cordifolia* on the cassava and plantain, used as test samples in making the varieties of fufu. The leaves of the *A*. *cordifolia* used are known to be nontoxic and highly considered safe for use in humans [54]. Moreover, a study by Ezeokeke et al. [55] indicated that the extract of *A*. *cordifolia* is nontoxic to the hematologic and renal systems. In this study, the phytoconstituents of *A*. *cordifolia* observed were alkaloids, flavonoids, phenols, saponins, tannins, and terpenoids. Various studies conducted on *A*. *cordifolia* revealed its significant hypoglycemic effect. Furthermore, *A*. *cordifolia* is also known to possess certain polyphenols (antioxidants) that have hypoglycemic properties [[30], [31], [32]]. Higher concentrations of flavonoids (polyphenols) are also associated with the inhibition of carbohydrate digestion by inhibiting key enzymes involved in digestion. They inhibit salivary α-amylase and α-glucosidase in the small intestinal brush border that is known to hydrolyze the terminal α-1, 4-linked glucose residues and glucose absorption and the stimulation of insulin secretion [56,57]. Glucose is absorbed across the intestinal enterocytes through specific transporters, and inhibition of the digestive enzymes or glucose transporters reduces the rate of glucose release and absorption in the small intestine and, thus, suppresses postprandial hyperglycemia [57].

Flavonoids are known to inhibit glucose absorption in the intestine by sodium-dependent glucose transporter 1, stimulate insulin secretion, and reduce hepatic glucose output. They improve acute insulin secretion and insulin sensitivity and the possible mechanisms that include a decrease in glucose absorption in the intestine and suppression of carbohydrate digestion, stimulation of insulin secretion from the pancreatic β cells, modulation of glucose release from the liver, activation of insulin receptors, and glucose uptake in the insulin-sensitive tissues [58]. Flavonoids also improve glucose uptake in peripheral tissues by modulating intracellular signaling [57] and can inhibit advanced glycation end-product formation [59].

Glycemic and insulin responses differ depending on the polyphenol–carbohydrate combination [57]. Flavonoids are known to reduce the peak and early-phase glycemic response and maintain the glycemic response in the later stages of digestion, leading to the reduction of postprandial and fasting hyperglycemia [60]. Again, flavonoids reduce peak insulin response and sustain the insulin responses, especially when consumed with carbohydrates [58].

Generally, flavonoids reduce blood glucose concentrations when consumed with a carbohydrate source, especially foods high in starch or glucose. The molecular structure of flavonoids allows them to interfere with starch digestion at the intestinal level and reduce glucose absorption into the blood [57]. Flavonoids have also been shown to inhibit digestive enzymes by preventing enzyme attack on starch, thereby reducing the amount of free glucose released and reducing glucose transport into the blood through the inhibition of specific glucose transporters in the intestinal lumen [56].

Other polyphenols, such as tannins, have protective effects of their capacity to activate antioxidant enzymes and enhance glucose uptake through mediators of the insulin-signaling pathways, such as phosphoinositide 3-kinase), p38 mitogen-activated protein kinase activation, and GLUT-4 translocation [61]. The reduction in blood glucose concentrations caused by tannins has been attributed to their ability to inhibit intestinal glucose absorption [62]. Tannins reduce glucose digestibility simply by inhibiting the digestive enzymes [63].

A physical observation during the processing of the fufu revealed a nonstarchy liquid produced when the cassava and plantain were cooked with the *A*. *cordifolia* compared with the usual starchy liquid that is produced when starchy carbohydrates are cooked. Thus, it may be concluded that a significant amount of starch was broken down during the cooking as suggested by Mohammeda et al. [64]. Although there was a colored liquid produced, it did not interfere with the original color nor the taste of the composited fufu from that of the noncomposited fufu.

The color of the cassava and plantain after cooking with the leaves of the *A*. *cordifolia* became deeper than their original color [65]. The leaves of the *A*. *cordifolia* gave a deeper version of the actual color of the substance used [66].

Sensory conversation with the participants on the 3 varieties of *A*. *cordifolia*–composited fufu consumed for appearance, texture, aroma, and taste affirmed pleasantness with the composited meals. The participants preferred all 3 varieties of *A*. *cordifolia*–composited fufu in all sensory characteristics. In the olden days, most people had the practice of boiling carbohydrate foods with the leaves of *A*. *cordifolia* to intensify the color of the foods, which made them more presentable and reduced the starch content of the food but they did not know that the leaves of the *A*. *cordifolia* had a relation with the GI of the food (F. C. Mills-Robertson, personal communication, 2021).

According to the study participants, there was no change of taste for either the noncomposited or composited fufu. Some of the participants felt nauseated after consumption of the reference food (glucose solution). The feedback from the participants were similar to those of the study by Brouns et al. [37] using white bread and glucose as reference materials in GI determination. Despite the observation, glucose remains the preferred reference material over white bread because of potential variations that could result from the preparation of white bread [67].

During the testing periods, evening meals before the testing morning had no restrictions. Granfeldt et al. [68] revealed in a study the effect of evening meals on the study subject who recorded the least glucose response for the test food due to the previous evening meal (oatmeal with higher fiber content). Some studies have also confirmed that meals previously taken before a morning meal can influence the glycemic response of that breakfast meal [68,69]. In this study, the FBG before any of the testing days for either the reference or the test food showed the least difference although the study subjects consumed a variety of evening meals. In this study, the study participants were asked to fast for 10–14 h before the commencement of the test.

In the study, the test fufu could not be consumed alone, so they were served with the same quantity of light soup and the same size of salmon (fish) to ensure a null difference that could be attributed to the soup and fish served with the test fufu. The light soup was a better serving choice because it had a less impact on the fiber and available carbohydrate portions that were necessary parameters in measuring GI.

Protein hydrolysate in meals exhibited an intense significant effect on insulin and glucagon responses relative to the type and quantity served per the body weight of the individual [70]. In a study where type II diabetic subjects were given 50 g glucose with different 25 g of proteins, which included egg white, the same level of glucose response was found with either glucose ingestion alone or glucose ingested with egg white [71]. Because the egg white did not affect the glucose response, it indicated the fact that the type and quantity of protein are necessary to influence glucose response [72].

In this study, the quantity and type of protein served were the same for the test foods and could not have significantly affected the glucose response. The salmon served had no available carbohydrate portion and the light soup had relatively small available carbohydrates, which provided an nonsignificant increase in the glucose response.

It may be concluded from this study that *A*. *cordifolia* can reduce the GI of fufu prepared from cassava, plantain, or a combination of cassava and plantain. There were significant differences between the noncomposted and the *A*. *cordifolia*–composited fufu. The phytoconstituents of *A*. *cordifolia* included flavonoids and tannins, which might have had reduced the GIs.

### Author contributions

The responsibilities of the authors were as follows – ETO, FCMR, MAT: designed research; ETO: conducted research, provided essential materials, and analyzed data; ETO, FCMR: wrote the paper; ETO, FCMR, MAT: had primary responsibility for final contents; and all authors: read and approved the final manuscript.

### Conflict of interest

ETO, FCMR, MAT reports administrative support, equipment, drugs, or supplies, statistical analysis, travel, and writing assistance and has patent Ethical Clearance issued to CHRPE/AP/119/21. FCMR, MAT reports a relationship with Kwame Nkrumah University of Science and Technology that includes: employment.

### Funding

The authors reported no funding received for this study.

### Data availability

Data described in the manuscript, code book, and analytic code will be made publicly and freely available without restriction at https://drive.google.com/drive/folders/1T33d0zyGtzP8i6V2Is3FaTHO34IvE3rU?usp=sharing.
